# Inhibitory role of supraspinal P2X_3_/P2X_2/3 _subtypes on nociception in rats

**DOI:** 10.1186/1744-8069-2-19

**Published:** 2006-06-05

**Authors:** Masato Fukui, Takayuki Nakagawa, Masabumi Minami, Masamichi Satoh, Shuji Kaneko

**Affiliations:** 1 Department of Molecular Pharmacology, Graduate School of Pharmaceutical Sciences, Kyoto University, Kyoto 606-8501, Japan; 2 Department of Pharmacology, Graduate School of Pharmaceutical Sciences, Hokkaido University, Sapporo 060-0812, Japan; 3 Yasuda Women's University, Hiroshima 731-0153, Japan

## Abstract

Extracellular ATP is known to mediate synaptic transmission as a neurotransmitter or a neuromodulator via ionotropic P2X and metabotropic P2Y receptors. Several lines of evidence have suggested that ATP facilitates pain transmission at peripheral and spinal sites via the P2X receptors, in which the P2X_3 _subtype is considered as an important candidate for the effect. Conversely, we previously found that the activation of supraspinal P2X receptors evoked antinociception. However, the subtypes responsible for the antinociception via supraspinal P2X receptors remain unclear. In the present study, we showed that intracerebroventricular (i.c.v.) pretreatment with A-317491 (1 nmol), the novel non-nucleotide antagonist selective for P2X_3 _and P2X_2/3 _receptors, attenuated the antinociceptive effect produced by i.c.v. administered α,β-methylene-ATP (10 nmol), the P2X receptor agonist, in rats. Similarly, the abolishment of the P2X_3 _receptor mRNA in the brainstem by repeated i.c.v. pretreatments with antisense oligodeoxynucleotide for P2X_3 _gene once a day for 5 consecutive days diminished the antinociceptive effect of α,β-methylene-ATP. Furthermore, i.c.v. administration of A-317491 (1 and 10 nmol) significantly enhanced the inflammatory nociceptive behaviors induced by the intraplantar injection of formalin and intraperitoneal injection of acetic acid. Taken together, these results suggest that supraspinal P2X_3_/P2X_2/3 _receptors play an inhibitory role in pain transmission.

## Findings

Extracellular ATP has been established as a signaling molecule that mediates diverse biological effects via P2 purinoceptors (P2X_n _and P2Y_n_) in both the peripheral and central nervous systems [1,2]. A body of evidence indicates that ionotropic P2X receptors are involved in both peripheral and spinal pain transmission [3-5]. Of the seven P2X receptors identified to date, the expression of the P2X_3 _receptors appear selective for a subpopulation of small-diameter dorsal root ganglion neurons, which are probably associated with nociception [6-8]. In vivo studies have provided evidence that the activation of P2X receptors, especially the P2X_3 _subtype and P2X_2/3 _heteromer, contributes to an acute nociceptive behavior, hyperalgesia and allodynia [9-12]. These observations support the idea that the P2X_3 _and P2X_2/3 _receptors play a crucial role in facilitating pain transmission at the peripheral and spinal sites. On the other hand, at the supraspinal level, we previously reported that the intracerebroventricular (i.c.v.) administration of ATP and P2X receptor agonists produced mechanical and thermal antinociception in rats, suggesting that supraspinal P2X receptors play an inhibitory role in nociceptive transmission [13-15]. However, it remains unclear what subtypes of supraspinal P2X receptors are involved in the antinociceptive effect. In the present study, to clarify the involvement of P2X_3_/P2X_2/3 _receptors, we investigated the effect of i.c.v. pretreatment with A-317491, the novel non-nucleotide antagonist selective for P2X_3_/P2X_2/3 _receptors [16,17] and down-regulation of P2X_3 _receptors in the brain by antisense oligodeoxynucleotide (A-ODN) in rats. Furthermore, to determine the roles of the endogenous purinergic system through supraspinal P2X_3_/P2X_2/3 _receptors in the exacerbated pain states, we examined the effect of A-317491 administered i.c.v. on the inflammatory nociceptive behaviors induced by chemical agents.

Male Sprague-Dawley rats weighing 180–250 g were used. The experiments were conducted in accordance with the ethical guidelines of the Kyoto University animal experimentation committee, and the guidelines of the Japanese Pharmacological Society. The surgery for i.c.v. administration was conducted according to our previous report [13]. After surgery, the animals were returned to cages and housed individually, being allowed to recover for 5 to 7 days until the experiments started. The i.c.v. injection was carried out in a volume of 5 μl at a constant rate of 5 μl/30 sec.

The 20-base A-ODN endcapped with phosphorothioate linkages (Nisshinbo Inc, Japan) were designed according to the primary sequence of the rat P2X_3 _receptor cDNA (X91167) as previously described [9]. The sequence of A-ODN was 5'-T*G*AA*GAAGTCTGATATAC*A*G-3', while the sequence of sense oligodeoxynucleotide (S-ODN) served as a control was 5'-C*T*GTATATCAGACTTCTT*C*A-3' (* as phosphorothioate). A-ODN and S-ODN were dissolved in PBS. I.c.v. injection of PBS (5 μl), A-ODN (1 nmol/5 μl), or S-ODN (1 nmol/5 μl) was carried out once a day for 5 consecutive days, and the behavioral assessment was conducted on next day of the last injection. After the behavioral test, the brain was removed, and the brainstem sample was dissected. Then, the mRNA levels for P2X_3 _receptor and β-actin in the brainstem were analyzed using an RT-PCR method.

The antinociceptive effect of α,β-methylene-ATP administered i.c.v. was measured by the paw pressure test, as previously described [13,14]. Briefly, using an analgesimeter (Ugo Basile, Milan, Italy), the cuneate piston was put on the right hind paw and the pressure was loaded at a rate of 32 g/sec. The pressure that elicited paw withdrawal behavior, was determined as the nociceptive threshold. After several habituation procedures, α,β-methylene-ATP (Sigma, St. Louis, MO) dissolved in PBS was administered i.c.v. at a dose of 10 nmol, and the nociceptive threshold was measured 5 min after the i.c.v. administration according to our previous reports [13,14]. Nociceptive behaviors evoked by inflammatory stimuli using formalin and acetic acid were assessed. For the measurement of formalin-induced nociceptive behavior, scoring of nociceptive behavior started immediately after the intraplantar (i.pl.) injection of 2% formalin at a volume of 100 μl into the right hindpaw. The nociceptive behaviors were observed for 60 min and were quantified using a rating scale method by assigning weights to the following categories: the injected paw was elevated, and was not in contact with the floor (weight = 1); and the injected paw was licked, bitten, or shaken (weight = 2). The time an animal spent in each behavioral category was multiplied by the category weight (1 or 2), summed, and then divided by the total test session period (300 sec). For the measurement of acetic acid-induced nociceptive writhing behavior, counting the number of typical writhing behaviors, characterized by a wave of contraction of the abdominal musculature followed by extension of the hind limbs, started immediately after the intraperitoneal (i.p.) injection of 2% acetic acid at a volume of 1 ml. The writhing behavior was observed for 60 min and the number of occurrences every 5 min was evaluated.

The statistical significance was calculated using one-way or two-way analysis of variance (ANOVA), followed by the Bonferroni *post hoc *test. Differences at *P *< 0.05 were considered significant.

**Figure 1:**
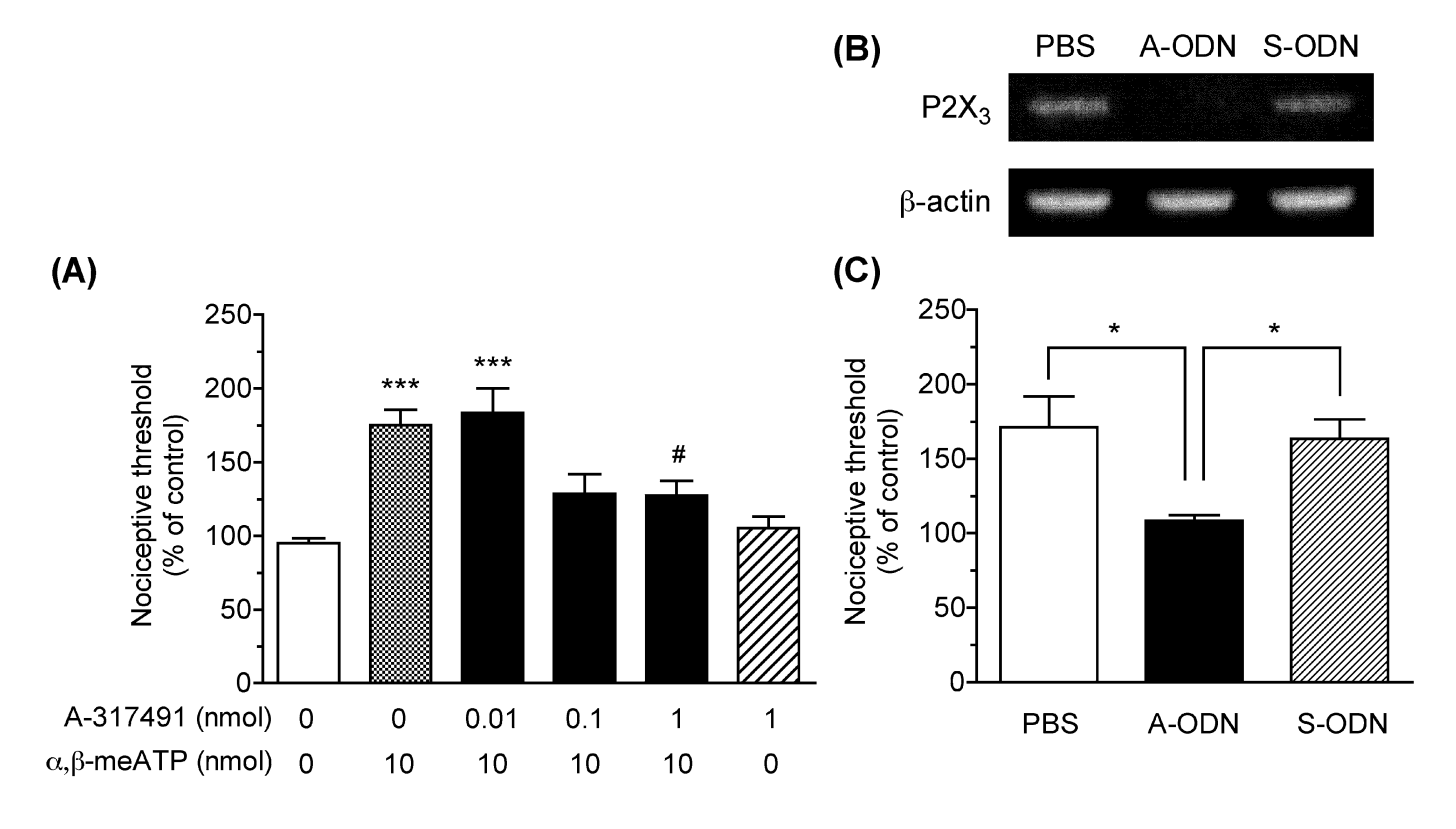
**The involvement of supraspinal P2X_3_/P2X_2/3 _receptors in the antinociception produced by i.c.v. administration of α,β-methylene-ATP**. The nociceptive thresholds at 5 min after the i.c.v. administration of α,β-methylene-ATP (10 nmol) were compared in the paw pressure test. The nociceptive threshold of each animal before the i.c.v. administration served as the control value (100%), and the values are presented as the means of the % of the control ± S.E.M. (A) The effect of i.c.v. pretreatment with A-317491. α,β-Methylene-ATP or PBS was administered i.c.v. 15 min after i.c.v. pretreatment with A-317491 (0.01–1 nmol) or PBS. ***P < 0.001 vs PBS-PBS group, ^#^*P *< 0.05 vs PBS-α,β-methylene-ATP group (*n *= 5–8). (B) RT-PCR analyses of the mRNA expression levels for P2X_3 _receptor (upper) and β-actin (bottom) in the brainstem after repeated i.c.v. pretreatments with PBS, P2X_3 _A-ODN (1 nmol), and P2X_3 _S-ODN (1 nmol). (C) The effects of repeated i.c.v. pretreatments with PBS, P2X_3 _A-ODN and P2X_3 _S-ODN on the antinociception by i.c.v. administered α,β-methylene-ATP. *P < 0.05 (*n *= 5–6).

The effect of i.c.v. pretreatment with A-317491 (Sigma) on the antinociceptive effect produced by i.c.v. administered α,β-methylene-ATP was examined (Fig. 1A). I.c.v. administration of α,β-methylene-ATP (10 nmol) at 15 min after i.c.v. pretreatment with PBS produced a significant antinociceptive effect, compared with the PBS-administered group (*P *< 0.001). The antinociceptive effect was dose-dependently and significantly reduced by i.c.v. pretreatment with A-317491 (*F*_5,29 _= 9.43, *P *< 0.001). The significant inhibitory effect of A-317491 was observed at a dose of 1 nmol, compared with PBS-pretreated group (*P *< 0.05). A-317491 (1 nmol) itself had no effect on the nociceptive threshold.

In the rat i.c.v. pretreated repeatedly with PBS and P2X_3 _S-ODN, the expression of P2X_3 _receptor mRNA in the brainstem was observed, while it was diminished by the repeated i.c.v. pretreatments with P2X_3 _A-ODN (Fig. 1B). In the rat pretreated with PBS and P2X_3 _S-ODN, i.c.v. administration of α,β-methylene-ATP (10 nmol) produced an antinociceptive effect, and there was no difference between them. The antinociceptive effect was significantly reduced by the repeated i.c.v. pretreatments with P2X_3 _A-ODN (*F*_2,14 _= 6.61, *P *< 0.01), compared with the PBS-pretreated (*P *< 0.05) and P2X_3 _S-ODN-pretreated (*P *< 0.05) groups (Fig. 1C).

**Figure 2:**
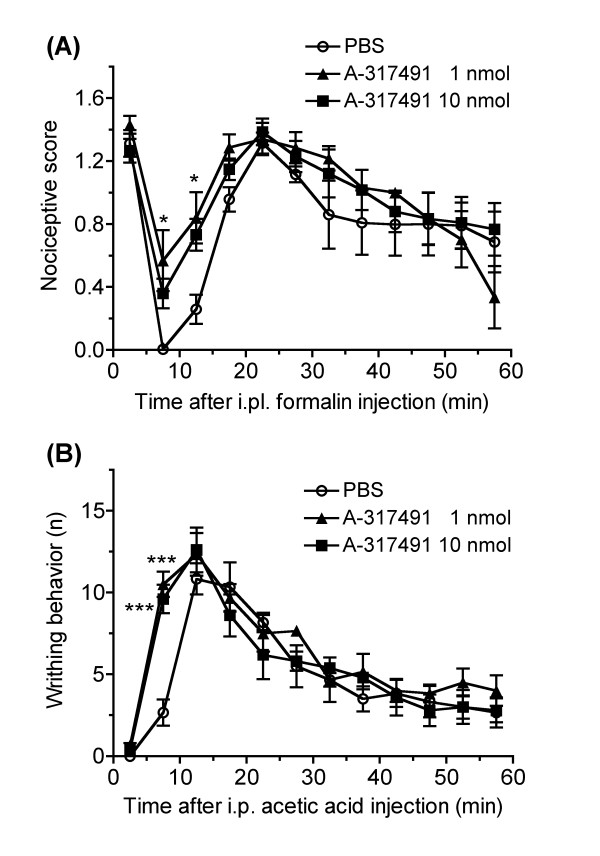
**The effect of i.c.v. administration of A-317491 on the inflammatory nociceptive behaviors**. (A) For the measurement of formalin-induced nociceptive behavior, the scoring of nociceptive behavior started immediately after the i.pl. injection of formalin into the right hindpaw. The nociceptive behaviors were observed for 60 min and were quantified using a rating scale method by assigning weights for each 5 min. (B) For the measurement of acetic acid-induced nociceptive writhing behavior, counting the number of typical writhing behaviors started immediately after the i.p. injection of acetic acid. The writhing behavior was observed for 60 min and the number of occurrences every 5 min was evaluated. The i.c.v. administration of A-317491 (1 and 10 nmol) or PBS was performed 5 min before the i.pl. injection of formalin or i.p. injection of acetic acid. The values are presented as the means ± S.E.M. *P < 0.05, ***P < 0.05 vs PBS group (*n *= 5–6).

To clarify the roles of supraspinal P2X_3_/P2X_2/3 _receptors in inflammatory pain, we examined the effect of the i.c.v. administration of A-317491 on inflammatory nociceptive behaviors induced by i.pl. formalin and i.p. acetic acid (Fig. 2). The i.pl. injection of formalin produced characteristic biphasic nociceptive behaviors. The i.c.v. administration of A-317491 (1 and 10 nmol) 5 min before the formalin injection significantly increased the nociceptive behaviors (*F*_2,168 _= 6.07, *P *< 0.01). Similarly, the number of acetic acid-induced writhing behaviors was significantly increased by the i.c.v. administration of A-317491 (1 and 10 nmol) 5 min before the acetic acid injection (*F*_2,168 _= 6.73, *P *< 0.01).

The present study provides evidence that P2X_3 _and/or P2X_2/3 _receptors are responsible for supraspinal P2X receptor-mediated antinociception. A-317491, a selective antagonist for P2X_3 _and P2X_2/3 _receptors, produces antinociception at peripheral and spinal sites in rat models of inflammatory and neuropathic pain[16-19]. Conversely, we found that the acute i.c.v. injection of A-317491 blocked the antinociceptive effect of α,β-methylene-ATP, which is selective for P2X_1_, P2X_3 _and P2X_2/3 _receptors, suggesting that the activation of supraspinal P2X_3_/P2X_2/3 _receptors exerted the antinociceptive effect [16,17]. The P2X_3 _subtype expressed in the brain is primarily distributed throughout the rat hindbrain, including the nucleus of the solitary tract, medial vestibular nucleus, medial and lateral parabrachial nuclei, rostral ventrolateral medulla and locus coeruleus (LC) [20,21], while the P2X_2 _subtype is widely distributed in the brain, including the hindbrain [22]. Therefore, it is assumed that the decreased effect of α,β-methylene-ATP-induced antinociception by repeated i.c.v. pretreatments with P2X_3 _A-ODN is attributed to the decreased expression of P2X_3 _receptors in the hindbrain. In the the nucleus of the solitary tract, the ultrastructural study showed that P2X_3_-immunoreactive boutons synapsed on dendrites and cell bodies, and had complex synaptic interaction with other axon terminals and vesiculated dendrites[20]. It is also described that α,β-methylene-ATP excites the neurons arising from the hindbrain, such as the LC [23,24]. We previously found that the bilateral microinjection of α,β-methylene-ATP into the LC produced robust antinociception, which was inhibited by co-injection with the P2X receptor antagonist PPADS[15], suggesting that the decreased level of P2X_3 _receptors in the LC by P2X_3 _A-ODN might have resulted in the blockade of α,β-methylene-ATP-induced antinociception.

There are several lines of evidence that endogenous ATP appears to play a facilitatory role in pain transmission at the peripheral and spinal levels, which is consistent with the effect of the exogenous injection of ATP and P2X receptor agonists. For instance, it has been reported that the intrathecal injection of PPADS attenuated the nociceptive behaviors induced by the i.pl. injection of formalin and capsaicin [25]. The intrathecal injection of P2X_3 _A-ODN also attenuated the hyperalgesia and allodynia after the nerve injury [9-11], however, the role of endogenous ATP in a supraspinal pain transmission has been unclear. Our present study found that i.c.v. administration of A-317491 deteriorated the inflammatory pain induced by formalin and acetic acid, suggesting that the endogenous ATP also plays an inhibitory role in pain transmission through P2X_3_/P2X_2/3 _receptors at the supraspinal level. The effect of A-317491 was rapidly normalized to the controlled level in both formalin- and acetic acid-induced inflammatory nociceptive behaviors. It is consistent with our previous findings that the antinociceptive effect by i.c.v. administered α,β-methylene-ATP was rapid and short lasting. The rapid effect of A-317491 is not probably due to the enzymatic degradation, as it is reported that A-317491 induces a long-lasting effect following systemic injection [16]. This might have resulted from the characteristic of the P2X_3 _receptor, which is known to be rapidly desensitized following its activation. Nevertheless, the i.c.v. injection of A-317491 did not affect the baseline nociceptive threshold in the paw pressure test (see Fig. 1A). These findings support the possibility that the inflammatory pain, but not acute pain, might stimulate the release of endogenous ATP into the extracellular space at the supraspinal level, as well as the spinal level[18,26,27].

Taken together, our findings suggest that the activation of supraspinal P2X_3_/P2X_2/3 _receptors produces antinociception. Since the effect at the supraspinal site is opposed to that at peripheral and spinal sites, further study might be needed to understand their role at the supraspinal site to develop potential analgesics via these receptors. Moreover, our results indicate that endogenous ATP at the supraspinal site plays an inhibitory role in the early stage of inflammatory pain.

## Abbreviations

ATP: adenosine 5'-triphosphate

A-317491: (5-({(3-phenoxybenzyl) [(1S)-1,2,3,4-tetrahydro-1-naphthalenyl]amino}-carbonyl)-1,2,4-benzenetricarboxylic acid)

PBS: phosphate-buffered saline

PPADS: pyridoxal-phosphate-6-azophenyl-2',4'-disulphonic acid

RT-PCR: reverse transcriptase-polymerase chain reaction

## Competing interests

The author(s) declare that they have no competing interests.

## Authors' contributions

MF carried out all the studies outlined in the manuscript, participated in the design of the studies and wrote the initial draft of the manuscript. TN conceived the study, participated in the design and the coordination of the study, performed statistical analysis, and wrote the final draft of the manuscript. MM participated in the design and coordination of the study. MS and SK supervised the experiments. All authors read and approved the final manuscript.
